# Some useful tips to detect Gift Authorship

**DOI:** 10.12669/pjms.36.6.3154

**Published:** 2020

**Authors:** Shaukat Ali Jawaid, Masood Jawaid

**Affiliations:** 1 Shaukat Ali Jawaid Chief Editor, Pakistan Journal of Medical Sciences Karachi - Pakistan; 2 Dr. Masood Jawaid Associate Editor Pakistan Journal of Medical Sciences Karachi - Pakistan

Gift authorship is considered a menace and intellectual corruption which is rampant all over the world but increasingly observed in developing world. Faculty members are under pressure to publish to meet some of the requirements of promotion. Hence either doing research under compulsion leads to a poor quality of manuscript; or to avoid it, some seek help and assistance of their colleagues to add their name on their manuscripts without having made any meaningful and intellectual contribution.

Authorship, it may be mentioned here, is the fulfillment of the responsibility to communicate research findings to fellow researchers for external evaluation which is also the primary basis for assessing a scientist’s contribution to the development of new knowledge.^1^ International Committee of Medical Journal Editors (ICMJE) has clearly defined as to who is eligible to be an author and they keep on revising their guidelines which was last revised in 2018. It lays down the four criteria to be fulfilled to qualify to be an author.^2^ However, in the real world it is almost difficult for all the listed authors to fulfill all the four criteria hence the editors have started to ask for individual contribution of all the listed authors. However, this too has not helped to check this menace of gift authorship. It is generally believed that the first author is the one who has done most of the research work or has the maximum intellectual contribution to the study as compared to the other listed authors. Regulatory authorities in different countries have different criteria to give credit to the listed authors. In Pakistan, now the first six listed authors get equal credit as per PM&DC criteria.

Gift authorship is defined as award of authorship to those who have no substantial intellectual contributions but their names are added just out of respect, friendship or some other benefits. Some feel it will ensure greater legitimacy.^3^,^4^ Some of the reasons for this wide spread menace are pressure or fear of the Heads of the Department, Supervisor or seniors who insist that their name should also be included in the authors. The young postgraduates, in their early days of professional career are much more vulnerable and they have no other option but to listen and fulfil the desire of their seniors. In some of the departments, there are unwritten rules that the name of the head of the department or head of the institution has got to be included in every paper being published. Some of these senior colleagues are extremely ambitious, selfish and they make all efforts to sabotage the authentic authorship list. This is despite the fact that they are fully aware of the ICMJE authorship guidelines.

Participants in one of the studies had given various reasons for attribution of authorship like writing the paper, seniority, and student’s supervisors. They looked at the gift authorship as helpful in maintaining relationship, a reward, or means to increase the manuscript’s credibility. Some also use it as an evidence of greater collaboration in research.^5^

Sometimes the number of publications are also used to claim research allowance, awards from the institution. Higher the h index, the greater are the chances for getting research allowance. Hence in order to achieve this target, some authors remaining in coveted positions keep on multiplying the number of their publications. These so called “Researchers” are the greatest beneficiary of Gift Authorship.

Great responsibility lies on the shoulders of Editors of medical journals who are supposed to uphold professional ethics and ensure that they do not become a part of this intellectual corruption by allowing Gift Authorship. However, it is not an easy task. It is extremely difficult to detect Gift Authorship. However, if one is sincere there are certain useful tips which can be used to detect Gift Authorship and discourage this practice. The prevalence of Gift authorship vary from country to country but it is generally believed that if a manuscript has more than four or five authors, it does include some gift authorship. If the number of listed authors is more than eight to ten, the chances of gift authorship also increases although there are some exceptions. This does not apply to multicenter studies and clinical trials which are undertaken at numerous centers and in many countries.^5^ All the listed authors are supposed to read the final version of the manuscript before submission to the journals for publication. Hence, if the Editors find some glaring inaccuracies, mistakes, it will strengthen the belief of Gift authorship.

Some other useful Tips which can help the editors to identify Gift authorship or those who benefit from gift authorship the most include the following:

1. More publications per year with a long list of authors.
2. If someone is the last author on too many publications.
3. The spouse’s name is often included in several publications.
4. Lack of formal education and training in epidemiology and biostatistics.
5. General unethical and behaviour of the person.
6. If there is a difference in figures i.e. number of patients in the Abstract, Methods and Results section.
7. Some names of the listed authors are missing in the details of individual contribution of the listed authors.
8. Sometimes more names are included among the authors just to share the publication charges by the main author.
9. If a request is made after submission that the submitter forgot to mention the name of some author who had made lot of contributions to the study. Remember most often this is an after thought and the objective is to please someone for various reasons.
10. In the acknowledgment if the author used the word “I” am grateful to so and so for help and assistance but the number of listed authors on the manuscript is more than one, it is most likely the case of Gift Authorship.
11. Some of those listed as authors are also included in the acknowledgment.

Using some of the above guidelines and Tips, one can successfully detect the cases of Gift Authorship to some extent. Hence every effort should be made by the Editors of Biomedical Journals to arrest this fast spreading menace. Remember if someone gets promoted to Professor or Head of the Department based on these number of Gift papers, he or she is going to destroy the academic environment in the department. Such people are neither capable of helping, guiding their juniors, postgraduates nor will ever be inclined to promote research, academic activities in their units for fear of themselves being exposed. For the Editors it is of course a difficult task but they need to remain vigilant as their own and their journal’s credibility is also at stake.

## References

[1] National Institute of Health Guidelines for the Conduct of Research in the International Research Programme at NIH (2007). (Internet) National Institute of Health, Bethesda MD.

[2] http://www.icmje.org/recommendations/browse/roles-and-responsibilities/defining-the-role-of-authors-and-contributors.html.

[3] McKnecally M (2006). Put my name on the paper. Reflections on the ethics of authorship Thorac Cardiothoracic Surg.

[4] Claxton LD (2005). Scientific authorship. Part-2. History, recurring issues, practices and guidelines. Mutate Res.

[5] Jackie MS, Rogers WA, Israel M, Braunack-Mayers AJ (2010). Credit where credit is due?Regulation, research integrity and the attributions of authorship in the health sciences. Social Science and Medicine.

